# Two Decades of Multimodal Management of Colorectal Liver Metastases With Long‐Term Outcomes

**DOI:** 10.1111/ans.70658

**Published:** 2026-04-06

**Authors:** Ali Mohtashami, Prajwol Shrestha, Sharon Chen, Jean‐Luc Vrisakis, Nazim Bhimani, Nick Pavlakis, Stephen J. Clarke, Connie I. Diakos, Matthew Wong, Thomas J. Hugh

**Affiliations:** ^1^ Upper Gastrointestinal Surgical Department Royal North Shore Hospital St Leonards New South Wales Australia; ^2^ Department of Medical Oncology Calvary Mater Newcastle New South Wales Australia; ^3^ Faculty of Medicine and Health University of Sydney Sydney New South Wales Australia; ^4^ Northern Sydney Cancer Centre, Royal North Shore Hospital St Leonards New South Wales Australia; ^5^ GenesisCare St Leonards New South Wales Australia; ^6^ Northern Clinical School, University of Sydney Sydney New South Wales Australia; ^7^ Department of Medical Oncology Gosford Hospital New South Wales Australia

## Abstract

**Background:**

The management of patients with colorectal liver metastases (CRLM) is complex and requires a tailored approach based on disease extent, patient age, and comorbidities. Given the heterogeneity in presentation and evolving treatment algorithms, prognostic factors for long‐term survival following liver resection are contested. This study aimed to evaluate “real‐world” outcomes in patients with long‐term follow‐up after potentially curative liver resection at a tertiary Australian center.

**Methods:**

A retrospective analysis was conducted on prospectively collected data from patients who underwent first‐time liver resection for CRLM between 1998 and 2018. Standardized clinical, biochemical, and radiological follow‐up was undertaken. Multivariable Cox regression analysis was used to identify independent predictors of overall survival (OS), measured from the diagnosis of liver metastases to death or last contact.

**Results:**

Of 396 patients who underwent resection, 320 met the inclusion criteria. The median follow‐up was 123.3 months (95% CI: 110.5–131.0), median OS was 69.6 months (95% CI: 57.2–84.5), and the 10‐year OS rate was 33%; 5‐year and 10‐year disease‐free survival rates were 29% and 25%, respectively. Neoadjuvant chemotherapy was administered in 72% of patients, with 25% receiving targeted therapy in this setting. Postoperative (adjuvant or recurrence‐related) chemotherapy was given to 50% of patients, with 17% also receiving targeted therapy. Independent predictors of poor OS included older age, right‐sided primary tumours, large liver tumours, administration of locoregional liver therapy, and the use of chemotherapy ± targeted therapy in both the neoadjuvant and adjuvant settings.

**Conclusion:**

One‐third of patients who undergo liver resection for CRLM achieve long‐term survival. While multi‐modal therapy was commonly used, determining the specific contribution of non‐surgical treatments is challenging in a real‐world cohort where treatment paradigms evolved over time. This study provides rare 10‐year follow‐up data and reinforces the need for ongoing refinement of personalised management strategies for CRLM.

## 1 Introduction

Colorectal cancer (CRC) is the third most common malignancy worldwide and a major cause of cancer‐related mortality, and is responsible for approximately one million deaths each year [1]. In Australia, while age‐adjusted incidence rates have declined by one‐third since 2001, largely due to the National Bowel Cancer Screening Program, the absolute number of new cases is steady at around 15 000 annually due to population growth and ageing. As of 2024, the lifetime risk of CRC by age 85 is estimated at 1 in 21, and the disease accounts for nearly 1 in 10 new cancer diagnoses nationally [2].

At presentation or during follow‐up, around 50% of patients have or develop metastatic disease, most commonly to the liver. Colorectal liver metastases (CRLM) are a major source of morbidity and mortality, requiring a tailored, patient‐specific approach based on disease extent, comorbidities, and age. While multi‐modal therapy is important, hepatic resection provides the only potentially curative treatment. In well‐selected patients with favorable tumour biology, no extrahepatic spread, and adequate future liver remnant, 5‐year survival rates of 40%–60% are achievable [3, 4]. However, many patients are ineligible for immediate resection due to tumour burden or anatomical limitations.

Over the past 25 years, systemic therapy has progressed from fluorouracil/leucovorin monotherapy to combination regimens incorporating irinotecan and oxaliplatin (e.g., FOLFIRI, FOLFOX), improving response rates and survival [5, 6, 7]. The advent of targeted agents, particularly anti‐angiogenics like bevacizumab and epidermal growth factor receptor inhibitors such as cetuximab and panitumumab, has improved outcomes in biomarker‐selected patients [8], and in some patients, facilitated conversion from unresectable to resectable disease [9].

Surgical innovation has also enhanced the safety and scope of CRLM resection. Techniques such as portal vein embolization (PVE), hepatic vein occlusion, Associating Liver Partition and Portal vein Ligation for Staged hepatectomy (ALPPS), and two‐stage hepatectomy have expanded resectability and improved survival [10, 11, 12, 13]. The adoption of minimally invasive approaches, including laparoscopic and robotic‐assisted surgery, has further reduced morbidity and recovery time compared with open surgery [14, 15]. Locoregional therapies such as radiofrequency ablation (RFA), microwave ablation (MWA), laser‐induced thermotherapy (LITT), trans‐arterial chemoembolization (TACE), and selective internal radiation therapy (SIRT) have also broadened treatment options for patients with both resectable and unresectable disease, offering local disease control and in some cases survival benefit [16, 17, 18].

Despite these advances, optimal patient selection and long‐term outcomes following multimodal treatment are still evolving. There is ongoing debate regarding prognostic factors for long‐term survival following CRLM resection. While many studies have developed models to predict prognosis based on preoperative clinicopathological factors, these tools often show only modest correlation with outcomes and lose predictive power over time [19, 20]. Real‐world data, although inherently heterogeneous, reflect actual multidisciplinary team (MDT) decision‐making and the evolution of treatment strategies, and can provide valuable insights across a variety of clinical settings. Few surgical series have reported long‐term outcomes using contemporary MDT‐based treatment.

We hypothesized that patients treated for CRLM at a tertiary multidisciplinary center over the last two decades would show meaningful long‐term survival despite evolving treatment paradigms. The aim of this study was to evaluate long‐term outcomes in patients undergoing potentially curative treatment for CRLM at a single Australian tertiary institution.

## 2 Methods

### 2.1 Study Design and Patient Selection

This cohort study involved a retrospective analysis of a prospectively maintained database of patients undergoing first‐time liver resection for CRLM between 1998 and 2018 at a tertiary referral center in Australia. All patients were staged with a multi‐phase CT scan and PET scan, and management decisions were made through a multidisciplinary process. MRI was used routinely as an additional investigation from 2015 onwards. Resection criteria included disease amenable to complete removal with a likely negative margin while preserving sufficient future liver remnant volume to maintain postoperative liver function. The 2018 cut‐off was selected to ensure sufficient follow‐up time for assessing long‐term survival, including 10‐year outcomes or greater where possible. Ethics approval was provided by the Human Research Ethics Committee of the Northern Sydney Local Health District and the Central Coast Local Health District (RESP/18/76). The liver resection database was established in 1998 and includes all patients who underwent liver resection under the care of the senior author at Royal North Shore Hospital and North Shore Private Hospital, Sydney, New South Wales, Australia. The current study includes patients previously reported in studies focusing on surgical technique and perioperative outcomes [21, 22] but instead focuses on long‐term survival, recurrence patterns, and the impact of multimodal therapy. Only the first resection was included in patients who had multiple liver resections. Patients were excluded if they had squamous cell carcinoma metastases or had a liver biopsy only.

### 2.2 Patient Population and Data Collection

Demographic data (age, sex), radiological features (timing of metastases, laterality, tumour size), biochemical and histopathological factors (RAS, BRAF, microsatellite instability, mismatch repair status), treatment details, operative findings, and post‐operative outcomes were prospectively collected. RAS/RAF testing was uncommon before 2008 and was not routinely performed in our patients until 2010. Neoadjuvant chemotherapy and/or targeted therapy were defined as treatment delivered within 6 months before liver resection; adjuvant treatment as therapy given within 6 months after.

### 2.3 Surgical and Pathological Variables

Intra‐operative data included the diameter of the largest tumour, the number of tumours, the operative duration and technique, the extent of resection, blood loss, resection margin status, and tumour differentiation. Major resection was defined as the removal of at least three contiguous hepatic segments. An R0 margin was defined as tumour‐free ≥ 1 mm, and R1 as microscopic involvement or tumour within 1 mm of the cut surface.

### 2.4 Post‐Operative Outcomes

Post‐operative data included complications, length of stay, survival, recurrence, and receipt of further treatment (adjuvant chemotherapy, targeted therapy, liver ablation, and locoregional therapy [TACE or SIRT]). Complications were recorded according to the Clavien‐Dindo classification score up until 90 days post‐operatively, and then further grouped as either minor (Clavien‐Dindo ≤ II) or major (Clavien‐Dindo ≥ IIIa).

### 2.5 Definitions of Long‐Term Outcomes

Clinical, radiological, and biochemical follow‐up was performed routinely in all patients.

Overall survival (OS) was defined as the interval from the diagnosis of CRLM to death from any cause or last follow‐up, to capture the entire disease course, including patients who received pre‐operative systemic therapy.

OS calculated from the time of liver resection was also analysed to facilitate comparison with existing literature.

Disease‐free survival (DFS) was defined as the interval from the liver resection to the first documented recurrence or death, whichever occurred first.

### 2.6 Statistical Analysis

Categorical variables were presented as percentages and analyzed using the Chi‐squared test. Continuous variables were expressed as means and standard deviations and analyzed using the independent student *T*‐test, where the data were normally distributed. In cases of non‐normal distribution, median and range were reported, and variables were analyzed using the Mann–Whitney *U* test. OS and DFS were determined using the Kaplan–Meier survival analysis, and differences in survival were assessed with the log‐rank test. Cox proportional hazards univariable regression models were used to evaluate the association of relevant clinicopathological factors with prognosis. After testing for collinearity, all univariable analyses with a *p* value < 0.2 were included in the multivariable model. Backward elimination was used as a variable selection method. The proportional hazards assumption was verified using the Schoenfeld residuals, and the overall goodness of fit of the model was assessed using Cox‐Snell residuals. When the proportional hazards assumption failed, stratification of variables was attempted, and when this failed, a time‐varying component was introduced. To assess if the proportional hazards assumption was met for the time‐varying model, a likelihood ratio test was performed. Survival estimates were reported as hazard ratios (HRs) with 95% confidence intervals (CIs). For all analyses, a *p* value < 0.05 was considered statistically significant. All statistical analyses were performed using Stata BE for Windows version 17.1 (StataCorp, College Station, Texas, USA).

## 3 Results

### 3.1 Patient and Tumour Characteristics

A total of 320 patients underwent hepatic resection for CRLM. The demographic and clinicopathologic characteristics are summarised in Table 1. The median age was 65 years (range 29–85), and 63% were male. Right‐sided primary tumours were present in 29% of patients. Almost three‐quarters of patients (71%) received pre‐operative chemotherapy, most commonly oxaliplatin‐based doublets. Pre‐operative targeted therapies were administered in 22% of patients, predominantly bevacizumab. Among those tested, RAS mutations were identified in 48% and RAF mutations in 8%, with right‐sided primaries more likely to harbour these mutations (*p* < 0.001) or MSI high status (*p* = 0.032).

**TABLE 1:** Descriptive statistics.

	Total (*n* = 320)	Right sided (*n* = 92)	Left sided (*n* = 228)	*p*
Age in years, median (range)	65 (29‐85)	66 (33‐84)	64 (29‐85)	0.155
Gender				0.985
Male	202 (63.1)	58 (63.0)	144 (63.2)	
Female	118 (36.9)	34 (37.0)	84 (36.8)	
Pre‐operative chemo				0.881
Yes	228 (71.3)	65 (70.7)	163 (71.5)	
No	92 (28.7)	27 (29.3)	65 (28.5)	
Pre‐op chemo type				0.872
Capecitabine/5FU	72 (22.5)	23 (25.0)	49 (21.5)	
XELOX/FOLFOX	126 (39.4)	34 (37.0)	92 (40.3)	
XELIRI/FOLFIRI	15 (4.7)	5 (5.4)	10 (4.4)	
Other	15 (4.7)	3 (3.3)	12 (5.3)	
None	92 (28.7)	27 (29.3)	65 (28.5)	
Pre‐op biological				0.911
Bevacizumab	59 (18.4)	16 (17.4)	43 (18.9)	
Cetuximab or panitumumab	13 (4.1)	3 (3.3)	10 (4.4)	
None	248 (77.5)	73 (79.3)	175 (76.7)	
Mutation status (*n* = 86)				< 0.001
Wild type	38 (44.2)	3 (10.7)	35 (60.3)	
RAS mutant	41 (47.7)	19 (67.9)	22 (38.0)	
RAF mutant	7 (8.1)	6 (21.4)	1 (1.7)	
MMR (*n* = 117)				0.032
MSI normal	114 (97.4)	35 (92.1)	79 (100.0)	
MSI high	3 (2.6)	3 (7.9)	0 (0.0)	
Timing				0.727
Metachronous	151 (47.2)	42 (45.7)	109 (47.8)	
Synchronous	169 (52.8)	50 (54.3)	119 (52.2)	
Primary resection after liver				0.206
Yes	20 (6.3)	3 (3.3)	17 (7.5)	
No	300 (93.7)	89 (96.7)	211 (92.5)	
Length of operation in minutes, median (range)	180 (30‐510)	180 (60‐390)	180 (30‐510)	0.891
Hospital for liver resection				0.434
Public	149 (46.6)	46 (50.0)	103 (45.2)	
Private	171 (53.4)	46 (50.0)	125 (54.8)	
Chronic liver disease				0.628
Yes	5 (1.6)	2 (2.2)	3 (1.3)	
No	315 (98.4)	90 (97.8)	225 (98.7)	
Tumour site				0.800
Unilateral	210 (65.6)	58 (63.0)	152 (66.7)	
Bilateral	99 (31.0)	31 (33.7)	68 (29.8)	
Central	11 (3.4)	3 (3.3)	8 (3.5)	
Diameter of largest tumour in mm, median (range)	30 (0‐155)	30 (0‐150)	30 (0‐155)	0.255
No. of liver tumours				0.558
1	137 (42.8)	37 (40.2)	100 (43.9)	
2–3	128 (40.0)	41 (44.6)	87 (38.1)	
> 3	55 (17.2)	14 (15.2)	41 (18.0)	
Operative technique				0.348
Open	290 (90.6)	87 (94.6)	203 (89.0)	
Laparoscopic	21 (6.6)	4 (4.3)	17 (7.5)	
Lap to open	9 (2.8)	1 (1.1)	8 (3.5)	
Resection detail				0.533
Segmentectomy	80 (25.0)	25 (27.2)	55 (24.1)	
Sectionectomy	47 (14.7)	11 (11.9)	36 (15.8)	
Hepatectomy	86 (26.9)	22 (23.9)	64 (28.1)	
Extended Hepatectomy	42 (13.1)	16 (17.4)	26 (11.4)	
Non‐anatomical resection	65 (20.3)	18 (19.6)	47 (20.6)	
Major or Minor resection				0.659
Minor	187 (58.4)	52 (56.5)	135 (59.2)	
Major	133 (41.6)	40 (43.5)	93 (40.8)	
Pringle				0.509
Yes	279 (87.2)	82 (89.1)	197 (86.4)	
No	41 (12.8)	10 (10.9)	31 (13.6)	
Resection margin				0.143
R0	259 (80.9)	73 (79.3)	186 (81.6)	
R1	59 (18.5)	17 (18.5)	42 (18.4)	
R2	2 (0.6)	2 (2.2)	0 (0.0)	
Blood loss in mL, median (range)	175 (0‐3500)	150 (0‐1300)	200 (0‐3500)	0.708
Length of stay, median (range)	8 (1‐78)	7 (4‐78)	8 (1‐42)	0.406
Surgical complications				0.133
No	182 (56.9)	57 (62.0)	125 (54.8)	
Minor	80 (25.0)	16 (17.4)	64 (28.1)	
Major	58 (18.1)	19 (20.6)	39 (17.1)	
Surgical complications				0.244
No	182 (56.9)	57 (62.0)	125 (54.8)	
Yes	138 (43.1)	35 (38.0)	103 (45.2)	
Liver resection and comps				0.322
Minor resection and no comps	121 (37.8)	39 (42.4)	82 (36.0)	
Minor resection and comps	66 (20.6)	13 (14.1)	53 (23.2)	
Major resection and no comps	61 (19.1)	18 (19.6)	43 (18.9)	
Major resection and comps	72 (22.5)	22 (23.9)	50 (21.9)	
Tumour differentiation				0.949
Well	25 (7.8)	8 (8.7)	17 (7.5)	
Moderate	230 (71.9)	67 (72.8)	163 (71.5)	
Poor	15 (4.7)	4 (4.4)	11 (4.8)	
Not stated	50 (15.6)	13 (14.1)	37 (16.2)	
Multiple liver resections				0.012
Yes	41 (12.8)	5 (5.4)	36 (15.8)	
No	279 (87.2)	87 (94.6)	192 (84.2)	
Post‐operative chemo				0.288
Yes	152 (47.5)	48 (52.2)	104 (45.6)	
No	168 (52.5)	44 (47.8)	124 (54.4)	
Post‐op chemo type				0.343
Capecitabine/5FU	56 (17.5)	22 (23.9)	34 (14.9)	
XELOX/FOLFOX	56 (17.5)	15 (16.3)	41 (18.0)	
XELIRI/FOLFIRI	37 (11.6)	11 (12.0)	26 (11.4)	
Other	3 (0.9)	0 (0.0)	3 (1.3)	
None	168 (52.5)	44 (47.8)	124 (54.4)	
Post‐op biological				0.474
Bevacizumab	40 (12.5)	11 (12.0)	29 (12.7)	
Cetuximab or panitumumab	10 (3.1)	1 (1.1)	9 (4.0)	
None	270 (84.4)	80 (86.9)	190 (83.3)	
Post‐op liver ablation				0.220
Yes	27 (8.4)	5 (5.4)	22 (9.7)	
No	293 (91.6)	87 (94.6)	206 (90.3)	
Post‐op locoregional therapy				0.153
Yes	30 (9.4)	12 (13.0)	18 (7.9)	
No	290 (90.6)	80 (87.0)	210 (92.1)	
Recurrence after liver resection				0.438
No	97 (30.3)	25 (27.2)	72 (31.6)	
Yes	223 (69.7)	67 (72.8)	156 (68.4)	
Recurrence after liver resection				0.375
No	97 (30.3)	25 (27.2)	72 (31.6)	
Early (< 12 months)	137 (42.8)	45 (48.9)	92 (40.3)	
Late (≥ 12 months)	86 (26.9)	22 (23.9)	64 (28.1)	

### 3.2 Operative Details and Perioperative Outcomes

**FIGURE 1:**
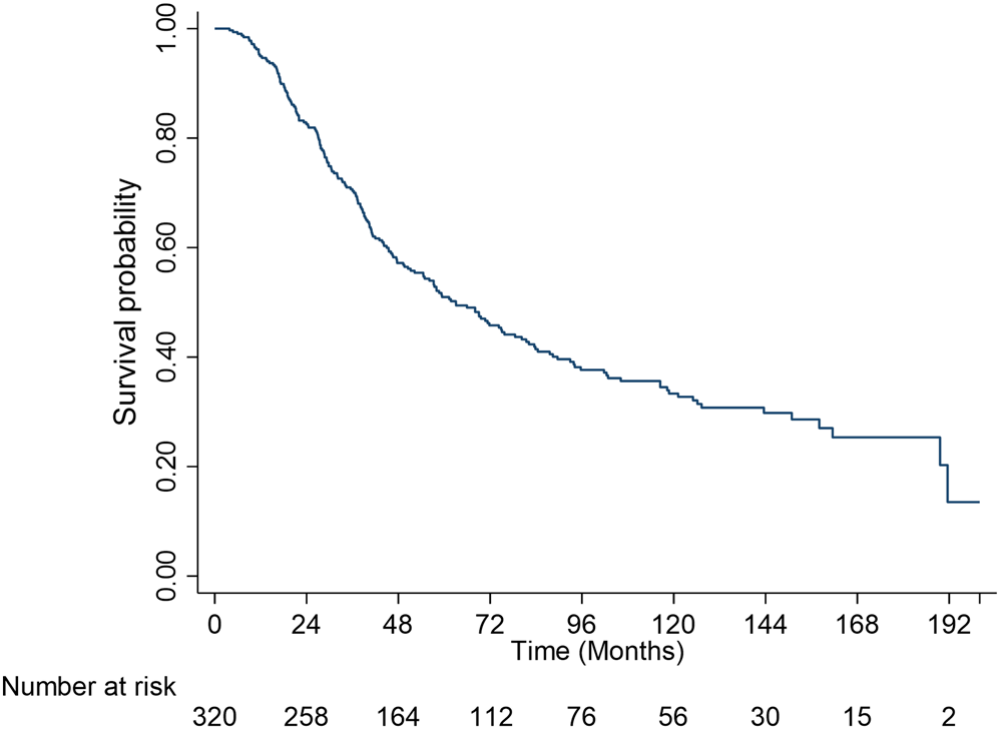
Overall survival. Median survival (from date of diagnosis to death): 63.05 months (95% CI: 52.24–75.73). Median follow‐up (from date of diagnosis to death): 123.27 months (95% CI: 112.66–131.02).

At the time of liver surgery, 53% of patients presented with synchronous disease. The median diameter of the largest metastasis was 30 mm (range 0–155), with 43% patients having a solitary lesion, 40% having 2–3 lesions, and 17% with three lesions. Bi‐lobar disease was present in 31% of patients. Most resections were performed as an open procedure (91%), and a major hepatectomy was undertaken in 42% of patients, including 13% who had extended liver resections. The median operative time was 180 min, with a median blood loss of 175 mL. An R0 resection margin was achieved in 81% of patients. The median post‐operative length of stay was 8 days (range 1–78). Overall, 43% of patients developed surgical complications, classified as major in 18% of patients (Figure 1). Right sided colon cancer patients were less likely offered multiple liver resections than left sided colon cancer patients (5.4% vs. 15.8%, *p* = 0.012).

Thirty‐day mortality was 0%, and 90‐day mortality was 0.6% (*n* = 2).

Only one patient had a combined liver resection and ablation. If patients had an ablation after surgery, it was for hepatic recurrence. Eight patients had a staged liver resection.

### 3.3 Multimodal Therapy

Pre‐operative chemotherapy was administered in 226 patients (71%), predominantly oxaliplatin‐based (FOLFOX) or irinotecan‐based (FOLFIRI) regimens, while 126 patients (39%) received post‐operative chemotherapy. Locoregional therapy was delivered in a subset of patients: 35 (11%) underwent post‐operative liver ablation with radiofrequency or microwave techniques, while 22 patients (7%) received post‐operative locoregional treatments (TACE or SIRT).

### 3.4 Survival Outcomes

The median OS from diagnosis of the CRLM was 63.1 months (95% CI 52.2–75.7), with a median follow‐up of 124 months.

The median OS calculated from the time of liver resection was 58.2 months (95% CI 46.4–71.9).

**FIGURE 2:**
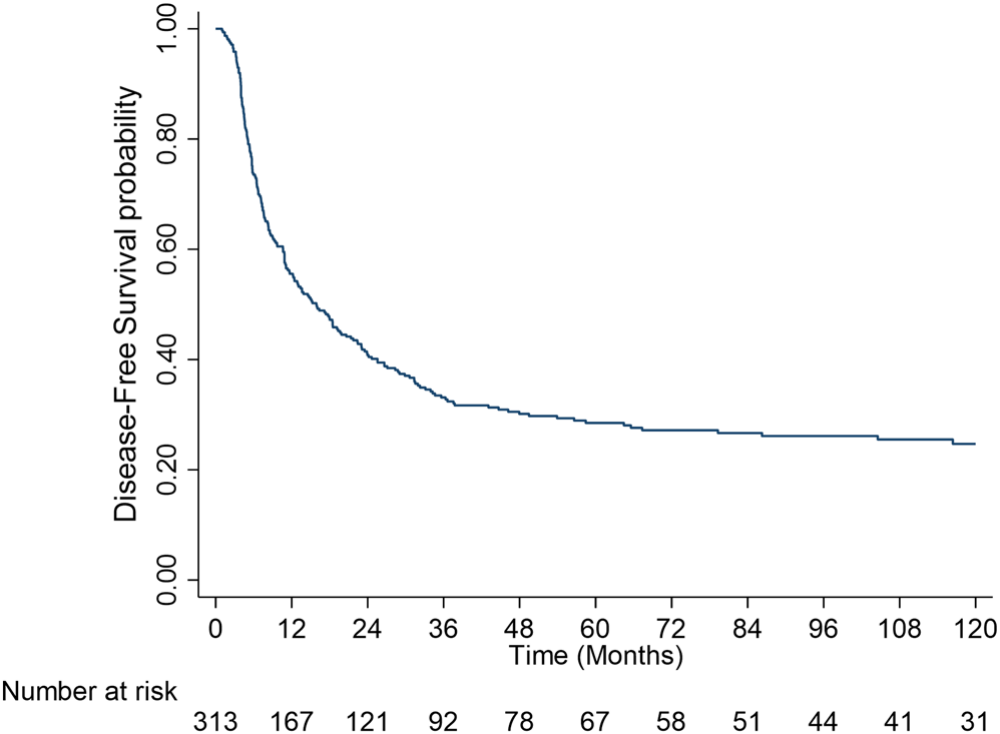
Median DFS (from liver resection to recurrence): 15.90 months (95% CI: 11.70–19.84).

The median DFS from liver resection to recurrence was 15.9 months (95% CI 11.7–19.8) as shown in Figure 2.

Five‐year and 10‐year OS rates were 51% and 33%, respectively.

Recurrence occurred in 70% of patients, with 43% relapsing within 12 months of surgery. Early recurrence carried a substantially higher hazard of death compared with late recurrence (HR 8.91 vs. 2.96, both *p* < 0.001). Most patients received systemic therapy, with 71% given pre‐operative chemotherapy and 39% post‐operatively, predominantly oxaliplatin‐ or irinotecan‐based doublets. Twenty‐two percent of patients had targeted therapy pre‐operatively, and 13% post‐operatively.

Chemotherapy alone, without targeted therapy, was associated with inferior OS and DFS, while the addition of targeted therapy (22% pre‐operatively, 13% post‐operatively, mainly bevacizumab) did not significantly influence long‐term outcomes. Locoregional therapies were used less frequently: 11% underwent post‐operative liver ablation, which was associated with short DFS but not OS, and 7% received selective internal radiotherapy or chemoembolization, which showed apparent short‐term benefit but worse long‐term survival in time‐varying models.

### 3.5 Prognostic Factors

Table 2 presents the univariable analyses for OS. Table 3 summarizes the multivariable model for OS.

**TABLE 2:** Univariable analysis for OS.

	HR	95% CI	*p*
Age			0.002
≤ 70 years	Reference		
> 70 years	1.57	1.18–2.10	
Age (1‐year increments)	1.02	1.01–1.03	0.007
Gender			0.166
Male	Reference		
Female	0.81	0.60–1.09	
Pre‐operative chemo			0.044
No	Reference		
Yes	1.38	1.01–1.90	
Pre‐op biological			0.972
None	Reference		
Bevacizumab	1.04	0.72–1.50	
Cetuximab or panitumumab	1.06	0.47–2.42	
Pre‐op biological			0.817
No	Reference		
Yes	1.04	0.74–1.48	
Pre‐op chemo + biologics			0.117
None	Reference		
Chemo + biologics	1.30	0.86–1.96	
Chemo only	1.42	1.02–1.97	
Mutation Status (*n* = 86)			0.004
Wild type	Reference		
RAS mutant	2.55	1.33–4.90	
RAF mutant	4.20	1.58–11.17	
MMR (*n* = 117)			0.485
MSI normal	Reference		
MSI high	1.66	0.40–6.84	
Timing			0.189
Metachronous	Reference		
Synchronous	1.21	0.91–1.60	
Sidedness			0.004
Right‐sided	Reference		
Left‐sided	0.65	0.48–0.87	
Primary resection after liver			0.958
No	Reference		
Yes	1.02	0.55–1.87	
Length of operation			0.008
≤ 180 min	Reference		
> 180 min	1.47	1.11–1.94	
Length of operation (1hr increments)	1.13	1.02–1.24	0.016
Hospital for liver resection			0.837
Public	Reference		
Private	0.97	0.73–1.28	
Chronic liver disease			0.355
No	Reference		
Yes	1.60	0.59–4.30	
Tumour site			0.058
Unilateral	Reference		
Bilateral	1.31	0.97–1.77	
Central	1.95	0.95–4.00	
Diameter of the largest tumour			0.003
≤ 30 mm	Reference		
> 30 mm	1.53	1.16–2.02	
Diameter of largest tumour (5mm increments)	1.05	1.02–1.07	< 0.001
No. of liver tumours			0.007
1	Reference		
2–3	1.34	0.98–1.83	
> 3	1.83	1.25–2.70	
Operative technique			0.023
Open	Reference		
Laparoscopic	0.39	0.17–0.88	
Resection detail			< 0.001
Non‐anatomical resection	Reference		
Segmentectomy	0.97	0.60–1.55	
Sectionectomy	1.38	0.83–2.29	
Hepatectomy	1.36	0.87–2.11	
Extended hepatectomy	2.59	1.60–4.20	
Major or minor resection			0.002
Minor	Reference		
Major	1.56	1.18–2.06	
Pringle			0.631
No	Reference		
Yes	1.11	0.72–1.70	
Resection margin			0.024
R0	Reference		
R1/R2	1.47	1.05–2.06	
Blood loss (100mL increments)	1.04	1.01–1.07	0.012
Length of stay (1‐day increments)	1.02	1.01–1.03	0.004
Length of stay			< 0.001
≤ 8 days	Reference		
> 8 days	2.16	1.63–2.86	
Surgical complications			< 0.001
No	Reference		
Minor	1.24	0.89–1.74	
Major	2.00	1.40–2.84	
Surgical complications			0.004
No	Reference		
Yes	1.50	1.14–1.99	
Liver resection and comps			0.001
Minor resection & no comps	Reference		
Minor resection & comps	1.35	0.91–2.01	
Major resection & no comps	1.40	0.94–2.10	
Major resection & comps	2.11	1.46–3.04	
Tumour differentiation			0.820
Well	Reference		
Moderate	0.92	0.55–1.55	
Poor	1.12	0.49–2.55	
Not stated	0.80	0.43–1.51	
Multiple liver resections			0.121
No	Reference		
Yes	0.71	0.46–1.09	
Post‐operative chemo			0.018
No	Reference		
Yes	1.40	1.06–1.86	
Post‐op biological			0.941
None	Reference		
Bevacizumab	0.93	0.60–1.44	
Cetuximab or panitumumab	1.03	0.42–2.51	
Post‐op biological			0.779
No	Reference		
Yes	0.94	0.63–1.41	
Post‐op chemo + biologics			0.017
None	Reference		
Chemo + biologics	1.12	0.73–1.71	
Chemo only	1.56	1.15–2.12	
Post‐op Liver Ablation			0.982
No	Reference		
Yes	0.99	0.61–1.62	
Post‐op locoregional therapy			0.001
No	Reference		
Yes	2.04	1.35–3.08	
Recurrence			< 0.001
No	Reference		
Yes	5.04	3.32–7.66	
Recurrence after liver resection			< 0.001
No	Reference		
Early (< 12 months)	8.91	5.72–13.88	
Late (≥ 12 months)	2.96	1.86–4.72	

**TABLE 3:** Multivariable analysis for OS.

	HR	95% CI	*p*
Age (10‐year increments)	1.24	1.07–1.43	0.004
Pre‐op chemo + biologics			0.032
None	Reference		
Chemo + biologics	1.47	0.92–2.36	
Chemo only	1.58	1.12–2.22	
Sidedness			0.018
Left‐sided	Reference		
Right‐sided	1.46	1.07–1.98	
Diameter of the largest tumour (5 mm increments)	1.05	1.02–1.07	< 0.001
No. of liver tumours			0.028
1	Reference		
2–3	1.81	0.34–9.51	
> 3	11.08	1.42–86.36	
Surgical complications			0.032
No	Reference		
Yes	5.05	1.15–22.13	
Post‐op chemo + biologics			0.032
None	Reference		
Chemo + biologics	1.02	0.65–1.62	
Chemo only	1.52	1.10–2.10	
Post‐op locoregional therapy (TACE or SIRT)			0.004
No	Reference		
Yes	0.019	0.001–0.287	
Time‐varying component			
No. of liver tumors			0.185
1	Reference		
2–3	0.92	0.59–1.44	
> 3	0.59	0.33–1.06	
Surgical complications			0.076
No	Reference		
Yes	0.69	0.46–1.04	
Post‐op locoregional therapy (TACE or SIRT)			0.001
No	Reference		
Yes	3.62	1.73–7.60	

*Note:* Disease‐free survival (from liver resection to first recurrence date).

On univariate analysis, adverse predictors of OS included age > 70, receipt of pre‐operative chemotherapy, right‐sided primary tumours, large tumor diameter, multiple metastases, major or extended hepatectomy, positive resection margins, prolonged operative time, increased blood loss, long length of stay, surgical complications, and receipt of adjuvant chemotherapy without biologics. RAS and RAF mutations were also associated with inferior survival.

Multivariate analysis confirmed that several factors independently predicted poor OS, including older age (HR 1.24 per 10 years, 95% CI 1.07–1.43, *p* = 0.004), right‐sided primary tumours (HR 1.46, 95% CI 1.07–1.98, *p* = 0.018), increasing tumour diameter (HR 1.05 per 5 mm, 95% CI 1.02–1.07, *p* < 0.001), multiple liver metastases (> 3 vs. 1, HR 11.08, 95% CI 1.42–86.36, *p* = 0.028) and surgical complications (HR 5.05, 95% CI 1.15–22.13, *p* = 0.032) at baseline. Time‐varying analysis showed that the adverse effects of multiple tumours (*p* = 0.185) and surgical complications (*p* = 0.076) diminished over time. Patients receiving pre‐operative chemotherapy (HR 1.58) or chemo‐biologics (HR 1.47) had significantly worse OS than those receiving no therapy (*p* = 0.032), and adjuvant chemotherapy without biologics also predicted inferior outcomes (HR 1.52, *p* = 0.032). Post‐operative locoregional therapy appeared protective at baseline (HR 0.019, *p* = 0.004) but conferred a detrimental long‐term effect (HR 3.62, *p* = 0.001).

For DFS, multivariate analysis identified larger tumour size (HR 1.04 per 5 mm, 95% CI 1.02–1.07, *p* < 0.001), multiple lesions (> 3 vs. 1, HR 2.15, 95% CI 1.34–3.45, *p* = 0.005), longer hospital stay (HR 1.35, 95% CI 1.03–1.78, *p* = 0.033) and adjuvant chemotherapy without biologics (HR 1.48, 95% CI 1.09–2.01, *p* = 0.038) as independent predictors of poorer outcomes (Table 4). Post‐operative liver ablation increased recurrence risk (HR 1.66, 95% CI 1.04–2.63, *p* = 0.033), as did post‐operative locoregional therapy (HR 1.80, 95% CI 1.15–2.85, *p* = 0.010).

**TABLE 4:** Multivariable analysis for DFS.

	HR	95% CI	*p*
Pre‐op chemo + biologics			0.100
None	Reference		
Chemo + biologics	1.55	1.01–2.39	
Chemo only	1.36	0.97–1.89	
Tumor site			0.406
Unilateral	Reference		
Bilateral	0.87	0.61–1.24	
Central	1.40	0.70–2.82	
Diameter of the largest tumor (5 mm increments)	1.04	1.02–1.07	< 0.001
No. of liver tumors			0.005
1	Reference		
2–3	1.45	1.05–2.01	
> 3	2.15	1.34–3.45	
Length of stay (1‐day increments)	1.35	1.03–1.78	0.033
Post‐op chemo + biologics			0.038
None	Reference		
Chemo + biologics	1.32	0.87–1.99	
Chemo only	1.48	1.09–2.01	
Post‐op liver ablation			0.033
No	Reference		
Yes	1.66	1.04–2.63	
Post‐op locoregional therapy (TACE or SIRT)			0.010
No	Reference		
Yes	1.80	1.15–2.82	

A total of 40 patients underwent repeat liver resections, including staged procedures. Consequently, 32 patients underwent repeat liver resection with curative intent for recurrent disease.

## 4 Discussion

This study presents long‐term outcomes following hepatic resection for CRLM over a 20‐year period at a single center. With a median follow‐up exceeding 10 years and a median OS of 63 months, the findings are favourable and align with international series from high‐volume centers [23]. More than 50% of patients were alive at 5 years and over one‐third at 10 years, highlighting the potential for long‐term survival despite metastatic disease. However, the recurrence rate was high at 70%, mostly occurring within the first year, emphasising the importance of tumour biology and timely multidisciplinary management.

The 10‐year OS observed is comparable to or better than previously published series. Buisman et al. reported an 18% 10‐year OS in a multicenter cohort [24], though their sample included patients with extrahepatic disease and incomplete resections. Rees et al. reported 21.8% 10‐year OS and 9.4% DFS across three UK centers, similar to our 11.1% DFS and slightly lower OS [25]. At the MSKCC, 22% of 725 patients achieved 10‐year OS and 20% were recurrence‐free [26]; while their recurrence‐free rate was higher, our cohort had greater OS (28.5%), possibly due to aggressive management of recurrent tumours. A more recent Argentinian study reported 22.3% 10‐year OS and 11.5% recurrence‐free survival among 200 patients [27], closely mirroring our own outcomes and confirming that long‐term cure is possible in about 10% of patients.

Several independent prognostic indicators were identified. Increasing age was associated with worse survival, with a 24% increase in mortality risk per decade. However, the prognostic role of age is still debated. A Chinese study found early‐onset colorectal cancer (< 50 years) was associated with worse OS, and that the impact of RAS mutations differed by age category [28]. In contrast, Toquero et al. concluded that age was not an independent prognostic factor when tumour burden, nutritional status, systemic therapy, and surgery were considered [29]. This supports the importance of tailoring treatment based on physiological rather than chronological age.

Tumour burden had a major impact on survival, with patients harboring more than three metastases experiencing an 11‐fold increased risk of death. Other groups have shown that volumetric measures like tumor size ratio (TSR) may provide additional prognostic value [30]. Right‐sided primary tumours were associated with inferior survival (HR 1.46), consistent with their higher rates of RAS/RAF mutations and previous literature [31]. This is reinforced by a 2020 systematic review that found improved survival in patients with left‐sided CRC likely due to differing embryological origins and molecular profiles [32].

Postoperative complications were significantly associated with worse outcomes (HR 5.05), as was postoperative chemotherapy alone (HR 1.52), potentially reflecting aggressive disease or patient frailty. Only 6.3% of our patients underwent liver‐first surgery, and sequencing did not significantly affect long‐term outcomes. Postoperative locoregional therapies, such as transarterial chemoembolisation (TACE) or SIRT, were associated with better early survival (HR 0.019).

A methodological strength of the current study was the use of time‐varying covariates to account for violations of the proportional hazards assumption. The adverse impact of multiple metastases and complications diminished over time, while the initial benefit of locoregional therapies reversed later (HR 3.62), likely reflecting eventual disease progression in a selected, aggressively treated cohort. While not powered to determine the standalone efficacy of locoregional therapies, our results are consistent with previous findings. The FOXFIRE‐SIRFLOX‐SIROX pooled analysis did not show a survival advantage for SIRT [18, 33, 34], but use of this regimen is often confounded by higher tumour burden. Similarly, the CLOCC trial found RFA improved progression‐free survival without affecting OS [35], The COLLISION trial demonstrated no significant difference in OS or local tumour progression between surgical resection and thermal ablation for small CRLM, supporting both modalities as effective treatment options when appropriately selected [36]. Accordingly, these data support treatment equipoise rather than superiority of resection alone in carefully selected patients.

The observed associations between systemic therapy or post‐operative locoregional treatments and inferior survival likely reflect treatment selection bias and underlying tumour biology rather than a causal detrimental effect of these therapies.

Patients requiring chemotherapy or locoregional therapy typically represent a subgroup with higher tumour burden, aggressive disease biology, or early recurrence, which cannot be fully adjusted for in retrospective analyses.

Systemic therapy use in our cohort reflects contemporary practice, with 71% of patients receiving pre‐operative chemotherapy and 39% receiving post‐operative chemotherapy. Chemotherapy without targeted therapy was associated with worse survival, while the addition of anti‐EGFR or anti‐VEGF agents did not significantly improve long‐term outcomes, consistent with recent literature [37]. This may reflect treatment selection bias, as patients with higher tumour burden or more aggressive disease biology are more likely to receive systemic or targeted therapy. Furthermore, while anti‐EGFR and anti‐VEGF agents are selected based on RAS status, updated guidelines such as those from ESMO recommend cetuximab only for left‐sided RAS wild‐type tumours and bevacizumab for right‐sided disease, even in RAS wild‐type patients [38]. Our findings may therefore reflect historical use of biologics in less selective or biologically aggressive contexts.

The association between systemic therapy and inferior survival, though counterintuitive, has several possible explanations. Retrospective analyses are vulnerable to unmeasured confounders, even after multivariate adjustment. Our results parallel the *New EPOC* trial, in which adding cetuximab to chemotherapy in resectable CRLM led to worse progression‐free survival (HR 1.48, *p* = 0.03), suggesting potential harm from overtreatment in some resectable cases. Additionally, toxicity‐related delays or impaired recovery could not be captured in this study, potentially contributing to the observed outcomes.

## 5 Conclusion

This study demonstrates that long‐term survival and cure are achievable in well‐selected patients undergoing hepatic resection for CRLM, particularly within the setting of multidisciplinary care. Unfortunately, recurrence is common, highlighting the need for careful patient selection and precision oncology approaches that integrate systemic, surgical, and locoregional strategies. While this study is limited by the retrospective nature of the analysis and the treatment heterogeneity, the real‐world and long‐term data presented here contribute to local and national benchmarks and reinforce the shift toward biology‐informed, individualised CRLM management.

## Author Contributions

**Ali Mohtashami:** writing – original draft, writing – review and editing. **Prajwol Shrestha:** data collection, investigation, validation, review and editing. **Sharon Chen:** data collection, investigation, validation. **Jean‐Luc Vrisakis:** data collection, investigation, validation. **Nazim Bhimani:** investigation, validation, software, formal analysis, data curation, methodology. **Nick Pavlakis:** validation, review and editing. **Stephen J. Clarke:** validation, review and editing. **Connie I. Diakos:** validation, review and editing. **Matthew Wong:** investigation, validation, review and editing. **Thomas J. Hugh:** concept, data collection, investigation, validation, writing – original draft review and editing.

## Funding

The authors have nothing to report.

## Conflicts of Interest

The authors declare no conflicts of interest.

## Data Availability

The data that support the findings of this study are available from the corresponding author upon reasonable request.
